# A comprehensive study on essential oil compositions, antioxidant, anticholinesterase and antityrosinase activities of three Iranian *Artemisia* species

**DOI:** 10.1038/s41598-022-11375-6

**Published:** 2022-05-04

**Authors:** Saba Shahrivari, Saeedeh Alizadeh, Kazem Ghassemi-Golezani, Elyas Aryakia

**Affiliations:** 1 Department of Horticultural Sciences, Faculty of Agriculture, University of Tabriz, Tabriz, Iran; 2 Department of Plant Ecophysiology, Faculty of Agriculture, University of Tabriz, Tabriz, Iran; 3 Plant Bank, Iranian Biological Resource Center (IBRC) (ACECR), Tehran, Iran

**Keywords:** Biochemistry, Plant sciences

## Abstract

*Artemisia* is one of the most diverse genera in the Asteraceae family. The genus is wildly distributed in Irano-Turanian habitats and includes 34 species in Iran. Here, for the first time the essential oil variability, antioxidants and anti-cholinesterase and anti-tyrosinase activities of extracts of three *Artemisia* species *(A. tournefortiana, A. khorassanica, A. haussknechtii)*, from different regions of Iran were evaluated. Based on GC–MS analyses, 81.84% to 98.70% of the total oils were identified. Cluster analysis grouped the studied populations in three different chemotypes. The highest and the lowest essential oil contents were observed in *A. khorassanica* and *A. haussknechtii* species, respectively. Camphor, en-in-dicycloether, 1,8-cineole and (*Z*)-*β*-farnesene were the dominant components of essential oil in investigated ecotypes. The results revealed that the total phenol content was higher in *A. tournefortiana* collected from Kerman and *A. haussknechtii* collected from Chaharmahal and Bakhtiari. However, the lowest phenol content was recorded for *A. haussknechtii* collected from Isfahan province. The highest flavonoids content was found in *A. tournefortiana* collected from West Azerbaijan and *A. khorassanica* collected from North Khorasan. The highest FRAP antioxidant activity was observed in *A. tournefortiana* (Kerman) and the lower amount was in *A. haussknechtii* collected from Kohgiluyeh and Boyer-Ahmad. The highest antioxidant activity by DPPH method was in *A. khorassanica* collected from South Khorasan and the lowest activity was in Isfahan's *A. haussknechtii.* The acetycholine esterase inhibitory activity was higher in *A. tournefortiana* collected from West Azerbaijan; and the lowest activity was in *A. haussknechtii* collected from Chaharmahal and Bakhtiari province. The highest tyrosinase inhibitory activity was in *A. khorassanica* collected from North Khorasan; and the lowest was in *A. haussknechtii* collected from Chaharmahal and Bakhtiari.

## Introduction

The genus *Artemisia* has a great distribution throughout the world and comprises 34 annual, biennial or perennial species throughout Iran^1^. Most of the *Artemisia* species are perennial, however about ten species are considered to be annual or biennial plants^2^. *Artemisia* species are mainly distributed in Asia, Europe and North America^3^. In Iran the distribution of *Artemisia* species is depends on various factors such as altitude and climatic conditions.

The genus *Artemisia* L. comprises medicinally important plants with valuable phytochemicals having a vast array of biological activities^3^. Over the years, more than 600 secondary metabolites belonging to different classes have been identified from *Artemisia* species, with valuable medical properties^4^. For thousands of years, the Chinese have been used *A. annua* as herbal tea preparation against malaria. *Artemisia* is considered to have a great potential for its biological activities, especially for treating viral infection and inflammation^5^. Artemisinin as the principal anti-malarial compound isolated from *A. annua* and the World Health Organization has recommended artemisinin-based combined therapies for the treatment of malaria^6^. Previous studies have been reported the insect’s repellent and insecticidal properties of the essential oil and the healing properties of *A. khorassanica* extract^7,8^. Recently, studies show that *Artemisia amygdalina* protects neurons through upregulation of Nrf2 pathway and may have the possibility to be a therapeutic agent for Alzheimer disease^9^. Ethanolic extract of *Artemisia haussknechtii* has antibacterial properties^10^.

Essential oils are among the important secondary metabolites of medicinal and aromatic plants. They have many usages in various industries and fields; from the pharmaceutical and cosmetic to the food and aromatherapy industries. Because of the various essential oil components, especially in leaves and flowers, some species of *Artemisia* genus possess a strong aroma. Many studies have shown that *Artemisia* species display significant intraspecific variations in the essential oil constituents. Various factors are involved in the diversity of essential oil compounds; such as pH, climatic factors and etc. In some cases, the variation in the volatile components of these plants may occur during plant ontogeny or growth at different altitudes^3^.

These days, researchers are paying attention to herbal medicines because of their fewer side effects; and there are some studies about medicinal plants inhibitory effects on acetylcholine esterase. For example, in a study concluded by Shekarchi et al*.*, relatively non-polar components of *F. persica var. persica* had AChEI activity^11^; or methanolic extract of *Mentha pulegium* had significant effect on the activity of this enzyme^12^. Acetylcholine is the neurotransmitter at synapses and within the central nervous system^13^. Alzheimer's disease (AD) is one of the most common forms of dementia. The reduction in acetylcholine synthesis is the main cause of AD. Therefore, increasing the cholinergic levels in the brain by inhibiting the biological activity of acetylcholinesterase (AChE) is one of the potential therapeutic strategies for preventing AD^14^.

Tyrosinase is an enzyme that is widely distributed in different organisms of plants and has an important role in the melanogenesis and enzymatic browning. Browning of fruits, fungi and vegetables is a common undesirable phenomenon. Tyrosinase is the main enzyme responsible for this enzymatic browning. Therefore, its inhibitors are attractive in medicinal industries as depigmentation agents and also as anti-browning compounds in food and agriculture industries^15^.

There is no any comprehensive research on three species of *A.tournefortiana, A.khorassanica,* and *A.haussknechtii*, so this research was designed to evaluate the variation in essential oil composition of these species as well as TFC, TPC, antioxidants, anti-cholinesterase and anti-tyrosinase activities of extracts.

## Materials and methods

### Reagents and standards

Folin and Ciocalteu’s phenol reagent, 2,2-Diphenyl-1-picrylhydrazyl Free Radical (DPPH), gallic acid, tannic acid, and *n*-alkanes were purchased from Sigma–Aldrich company (MO, USA). AlCl3, HCl, NaHCO3, HPLC grade methanol and GC grade *n*-hexane were purchased from Merck company (Darmstadt, Germany). Other chemicals and solvents were analytical grade and were purchased from Merck (Darmstadt, Germany).

### Plant materials, extraction and analysis of essential oil

The *Artemisia* species from Iranian provinces were collected and identified by the laboratory staff of Iranian Biological Resource Center (IBRC). All accessions were obtained under national and international guidelines and the plants were collected under the supervision and permission of Tabriz University and all authors comply with all the local and national guidelines.

**Figure 1:**
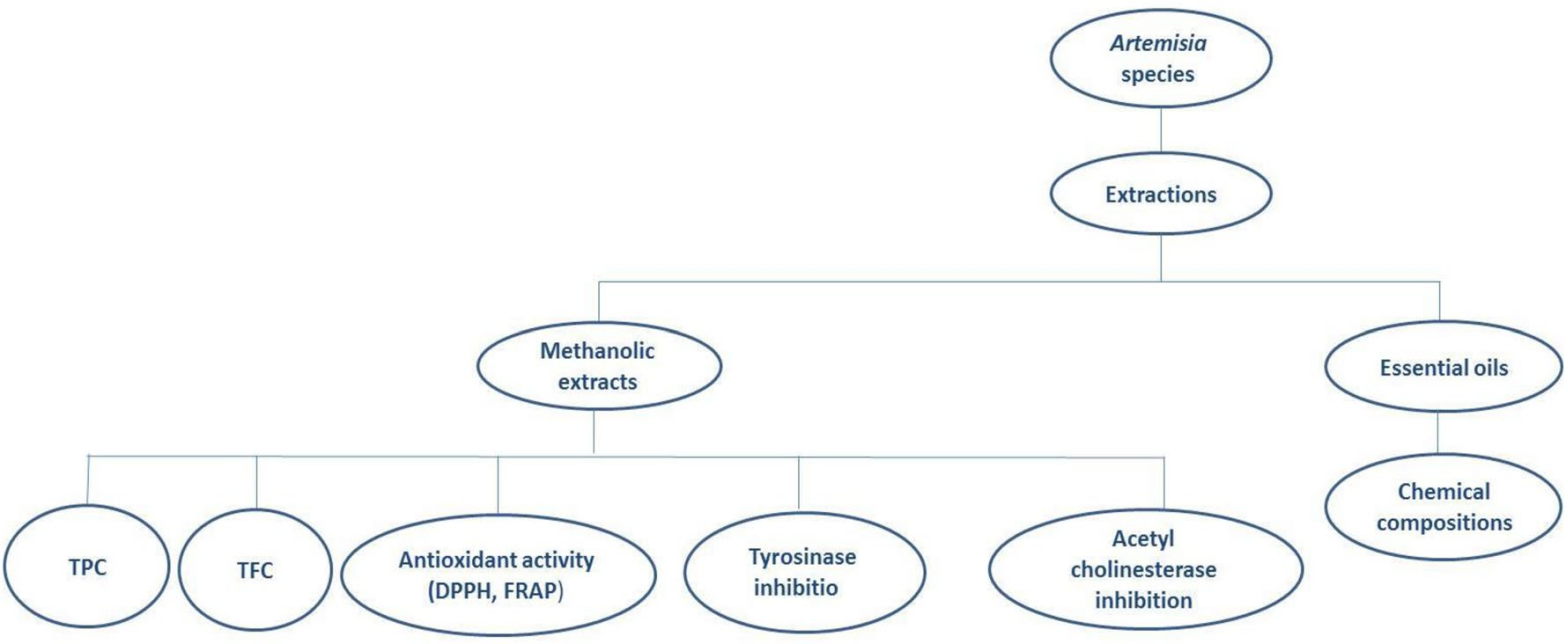
Schematic diagram of the experimental design.

Aerial parts of *A.tournefortiana* populations were collected from West Azerbaijan (Balolan village, IBRC No: IBRC P1000219), North Khorasan (Gifan toward Bojnourd, IBRC No: IBRC P1000632) and Kerman (Babzangi, S slopes of Hezar mt. IBRC No: IBRC P1006581) provinces. Aerial parts of *A.khorassanica* populations were collected from Semnan (Shahrod towards Azadshahr, IBRC No: IBRC P1000298), South Khorasan (Ghaen toward Sarayan, IBRC No: IBRC P1000750) and North Khorasan (Bojnourd, Baba Aman, IBRC No: IBRC P100639) provinces and aerial parts of *A.haussknechtii* populations were collected from Isfahan (Hanna, IBRC No: IBRC P1006441), Kohgiluyeh and Boyer-Ahmad (Sisakht, Bizhan pass, IBRC No: IBRC P1006481), and Chaharmahal and Bakhtiari (Malkhalifeh towards Lordegan, IBRC No: IBRC P1006501) provinces. Figure 1 shows a schematic diagram of the experiments.

Fifty grams of air-dried powdered plants (aerial parts) were subjected to hydro-distillation using a Clevenger type apparatus for 3–4 h (until the essential oil volume remained constant). Water to plant material ratio in hydro-distillation process was 10:1, the rate of distillation was about two drops per second.

The GC–MS analysis was performed on an Agilent, 19091S-443, USA instrument equipped with a HP-5MS column (30 m × 0.25 mm, film thickness 0.25 µm). The oven temperature was programmed as: 70 °C (held 3 min) raised to 120 °C at a rate of 10 °C/min (held 2 min), then heated up to 150 °C at a rate of 10 °C/min (held 2 min) and finally increased up to 240 °C at 7 °C/min and held 5 min in this temperature. Injector and transfer line temperatures were set at 230 and 240 °C, respectively. The essential oils were first diluted with *n*-hexane (1:100) and then 1 μL was injected into the GC–MS. The split ratio was 1:35. The essential oil components were identified by comparing their mass spectra with similar compounds from the WILEY275 and NIST 05 libraries. On the other hands, determination of arithmetic retention indices (RI) was investigated based on coherence of homologous series of hydrocarbons (Supelco, Bellefonte, USA) and comparing retention indices with those reported in the reference literature.

### Determination of total phenolic contents

Methanolic extracts (1:10) (methanol 80%) were used for total phenolic assay. For the extraction procedure, 1 g of powdered aerial parts samples were dissolved in 10 mL methanol: water 80:20 mixtures. The procedure was continued by shaking the samples for 72 h. After centrifugation, the supernatants were used for the assays. The method of Stankovic (2011)^16^ and Velioglu et al*.* (1998)^17^ with a slight change was applied to measure total phenolic content, using the folin-ciocalteu regent. To obtain the calibration curve 5 ml of folin-ciocalteu regent was dissolved in 50 cc distilled water and prepared the sodium bicarbonate solution (7.5%). So, different concentrations of gallic acid standards (100, 50, 25, 12.5 mg/L) by dissolving gallic acid in methanol 80%. Then, 3 ml of folin-ciocalteu solution and 3 ml of sodium bicarbonate solution were poured into the 15 mL centrifuge tubes and 100 µl of the extracts were added to this mixture. These solutions and standards were placed in water bath (45°) for 30 min. The control sample was mixture of 10% folin- ciocalteu and sodium bicarbonate7.5%. Finally, the absorbance was read at 765 nm with a spectrophotometer (Camspec—Model M550).

### Determination of total flavonoid contents

To measure the amount of total flavonoid, aluminum chloride )2%( was dissolved in methanol (80% in water). Then, we poured 1 ml of this solution and 1 ml of extract into the 15 mL centrifuge tubes, and incubated them at room temperature for 1 h. Quercetin standard solutions were made. At the end, the absorbance was read at 415 nm with spectrophotometer (Camspec—Model M550). The blank sample was aluminum chloride 2% solution^16^.

### Estimation of total antioxidant activity

#### DPPH method

DPPH method was used to measure the antioxidant content of the extracts. Briefly, 10 mg of DPPH was dissolved in 25 ml of methanol. Different concentration of the extracts (10, 20, 50, 100 μL) were prepared. 100 µl of sample (different concentrations) was dissolved in 200 µl of DPPH solution (0.2 mM); and then, the volume was increased to 400 µl with 80% methanol. After keeping in the darkness at room temperature for 30 min, the absorbance at 517 nm was investigated. Methanol and ascorbic acid were used as control and positive control samples, respectively. Ascorbic acid concentrations were prepared below 100 mg/kg (6.25, 12.5, 25, 50, and 100 mg/kg).

#### FRAP method

Benzie and Strain (1996)^18^ method was used to evaluate the antioxidant activity with some changes. Briefly, 300 mMol acetate buffer was prepared with pH 3.6 (3.1 gr sodium acetate was dissolved in 16 mL and the volume was increased to 1 L with distilled water), then 0.062 gr TPTZ (Tripyridyltriazine) was dissolved in 10 cc choloridric acid 40 mMol and 0.054 gr of FeCl_3_.6H_2_O was dissolved in 10 mL distilled water. To prepare FRAP solution, acetate buffer, TPTZ and iron chloride (10:1:1) were mixed together at 37 °C.

In this experiment 600μL of FRAP solution was added to 30μL methanolic extract and were kept at 37 °C for 8 min. The blank sample was FRAP solution. Fe2SO4.7H2O solution was prepared with different concentrations (500, 250, 125, 62.5 and 31.25 mg/kg) to draw a standard curve. Then, the absorbance was red at 593 nm with spectrophotometer (Camspec—Model M550).

### Acetyl cholinesterase inhibitory activity

For enzyme bioassay, the method of Ellman et al. (1961)^19^ with slight modifications was used. In this method, the inhibition of the acetylcholinesterase is determined by acetylcholine iodide, which is converted to thiocholine. In this method, 325 μl of 50 mM Tris buffer solution (pH8), 100 μl of plant extract in different concentrations (0.5–4 mg/ml) and 25 μl of enzyme solution (0.26 units per ml) was treated for 15 min at 25 °C. Then 75 μl of 15 mM acetylcholine iodide solution and 475 μl of 3 mM DTNB (Dithionitrobenzoic acid) solution were added. The absorbance of the sample was read at 412 nm^20^.

### Tyrosinase inhibitory activity

To measure the inhibitory activity of tyrosinase, the method of Zheng et al*.* (2013)^21^ with some modifications was used. The extracts were freshly prepared in DMSO (Dimethyl sulfoxide) solution at a concentration of 120 mg/mL and then diluted to lower concentrations (15–60 mg/ml) by DMSO solution, Then 50 µl of the test sample solution were dissolved in 450 μl of 0.05 mM sodium phosphate buffer (pH 6.8) and then 500 μl of L-tyrosine solution (0.1 mg/ml) was added. Finally, 500 μl of tyrosinase enzyme solution (200 units/ml) was added. DMSO and kojic acid solutions were used as control and positive control samples, respectively. The reaction mixture (1.5 mL) was mixed well with the vortex and the sample absorbance was read at 490 nm.

### Statistical analysis

Analysis of variance (ANOVA) and least significant difference (LSD) test at a 5% probability level was performed using SAS statistical software (version 9.1, SAS Inst., USA). Cluster analysis was done using IBM SPSS (SPSS, version 22, USA).

## Results and discussion

### Essential oils

The essential oil content of different ecotypes was between 0.02 and 0.1 ml (*A.haussknechtii*; *A.khorassanica*). In general, the average yield of essential oil was higher in *A. khorassanica* species and lower in *A. haussknechtii* species. Different ecotypes of *A. haussknechtii* species did not noticeably differ in essential oil yield. Few studies have been done on the composition of essential oils of these three species (*A. khorassanica, A. haussknechtii, A. tournefortiana*) in Iran. The major constituents of *A. khorassanica* essential oils were reported as 1,8-cineol, camphor, and davanone^22^. In another study, α-thujone, β-thujone and camphor were the main constituents of *A. khorassanica* essential oils^23^. Hadian et al. (2013)^24^ found that oxidated sesquiterpenes are the most essential oil constituents of the aerial parts of *A. khorassanica* during the flowering stage. Davanone, *p*-cymene, *Z*-citral, *β*-ascaridol and thymol were identified as the main components of *A. khorasanica* essential oil. The plants were collected from south of Khorasan (Saride village). In *A. tournefortina* (collected from India, Kashmir), cis-spiroether, *Z*-*β*-farnesene, trans-nerolidol and camphor were found to be the major constituents^25^. The main components of *A. tournefortiana* essence, collected from Firuzkuh (Iran), were E-thujone, sabinene and *β*-pinene^26^. In *A. haussknechtii* collected from Kermanshah, camphor, α-Terpineol, davana ether, and bornyl acetate were the major components^10^. Another study shows that the main components of the volatile oil of this species collected from north-west of Iran were 1,8—cineol, camphor, artemisia ketone, fragranol, yomogi alcohol and *β*- pinene^27^, which strongly supports the findings of our research.

Essential oils compounds were analyzed using GC–MS. According to the analysis of chromatograms, the essential oil compositions of *Artemisia* ecotypes are listed in Table 1. 81.84% of total oil for *A.tournefortiana* that collected from Kerman were identified. (*E*)-nerolidol (13.03%), (*Z*)-nerolidol (8.08%), camphor (7.69%) and (*Z*)-*β*-farnesene (7.65%) were the major components. 43.13% The identified compounds were sesquiterpenes and 34.90% of essential oil compounds were monoterpenes (Table 1).

**Table 1:** Essential oils compounds of *Artemisia* ecotypes.

	RI cal	RI REF^a^	E1	E2	E3	E4	E5	E6	E7	E8	E9
α-Pinene	930	932	–	–	–	–	–	0.126	–	–	0.42
3-Methyl-Cyclohexanol	930	935	–	–	–	–	–	–	0.154	–	–
Camphene	944	946	1.18	–	–	–	0.918	0.295	0.703	–	0.851
Benzaldehyde	950	952	–	–	–	–	–	0.368	–	–	1.179
Mesitylene	990	994	–	–	–	–	–	3.612	–	–	–
n-Decane	999	1000	0.11	1.44	–	0.114	–	–	6.732	–	–
δ-3-Carene	1006	1008	–	–	–	–	–	2.6	–	–	0.036
1,8-Cineole	1026	1026	–	–	–	–	22.917	7.358	2.099	–	16.569
ɣ-Terpinene	1056	1059	0.18	–	–	–	–	1.689	–	–	–
trans-Sabinene hydrte	1095	1098	–	–	–	–	–	–	7.59	–	–
Linalool	1098	1096	0.18	–	–	–	–	–	3.538	–	2.976
β-Thujone	1101	1101	0.89	–	–	–	–	–	1.002	–	–
Filifolone	1100	1109	–	–	–	–	–	4.707	–	–	–
Menth-2-en-1-ol < cis-ρ- >	1117	1118	–	–	–	–	–	0.481	–	–	–
Chrysanthenone	1120	1124	2.12	0.2	–	–	–	16.582	2.459	–	2.716
cis-β-Terpineol	1139	1140	6.02	–	–	–	–	–	0.067	–	–
Camphor	1140	1141	7.69	2.11	1.902	–	74.226	5.292	7.95	0.081	11.84
(2E,6Z)-Nonadienal	1151	1150	–	–	0.591	–	–	–	–	–	–
Borneol	1160	1165	–	–	–	–	–	2.054	–	–	16.139
Lavandulol	1161	1165	1.09	–	–	–	–	–	–	–	–
Terpinen-4-ol	1172	1174	–	–	–	–	–	2.525	–	–	–
Naphthalene	1176	1178	–	–	0.748	3.604	–	–	–	–	–
Verbenone	1206	1204	0.65	–	–	–	–	1.038	–	–	–
cis-Carveol	1226	1226	2.07	–	–	–	–	–	–	–	–
Pulegone	1230	1233	1.51	–	–	–	–	–	–	–	–
(E)-Ocimenone	1237	1235	–	–	–	–	–	0.984	–	–	–
Nerol	1238	1235	3.1	0.38	–	–	–	–	–	–	–
Chrysanthenyl acetate	1258	1261	–	–	–	–	–	1.251	6.68	–	3.317
Isopulegyl acetate	1274	1275	–	–	–	–	–	–	–	–	0.178
Isopiperitenone	1281	1285	0.56	–	–	–	–	0.772	–	–	–
Cyclopentadiene carboxylica cid	1283	1285	–	–	–	–	–	5.066	–	–	–
Bornyl acetate	1288	1287	3.17	–	0.369	–	–	1.056	2.348	–	4.559
Hydroxy citronellal	1289	1286	–	2.31	–	–	–	–	–	–	–
Thymol	1291	1289	–	–	–	–	–	2.685	–	–	–
Carvacrol	1299	1298	2.44	0.17	–	–	–	0.202	0.469	–	1.232
Azulene	1297	1298	–	1.67	–	3.115	–	–	–	–	–
Filifolide A	–	1996	–	–	–	–	–	1.921	–	–	–
Geranyl acetate	1381	1379	–	–	–	–	–	–	–	–	0.145
1-Tetradecene	1387	1388	0.92	0.47	0.235	–	–	0.499	0.84	–	0.41
(E)-Jasmone	1391	1390	–	–	–	–	–	4.864	–	–	–
trans-Caryophyllene	1413	1417	1.25	0.22	–	–	–	–	1.055	–	0.459
Benzimidazole	1428	1430	–	4.15	–	–	–	–	–	–	–
ɣ-Elemene	1434	1434	0.51	–	–	–	–	–	–	–	–
Aromadendrene	1438	1439	0.87	–	2.366	1.583	–	0.902	2.635	–	–
(Z)-β-Farnesene	1439	1440	7.65	1.68	19.463	–	–	–	0.127	–	–
9-epi-(E)-Caryophillene	1460	1464	1.19	0.12	–	–	–	–	–	–	–
ɣ-Gurjunene	1471	1475	–	–	–	–	–	–	0.183	–	–
Ledene	1495	1496	–	–	2.983	–	–	–	8.046	–	–
Davana ether	1496	1497	–	–	–	–	–	2.793	–	–	4.635
Methyl isoeugenol < (E)- >	1490	1491	–	–	–	–	–	–	2.113	–	–
Valencene	1497	1496	–	9.34	2.046	2.499	–	–	–	–	–
Bicyclogermacrene	1499	1500	0.43	0.21	–	–	–	–	–	–	–
β-Bisabolene	1507	1505	1.44	0.81	0.982	–	–	–	1.023	–	–
δ-Cadinene	1520	1522	2.27	–	–	–	–	–	–	–	–
(Z)-Nerolidol	1530	1531	8.08	15.25	0.988	5.242	–	–	–	–	–
α-Cadinene	1536	1537	0.3	0.99	0.345	–	–	–	–	–	–
α-Calacorene	1540	1544	0.67	–	–	–	–	–	–	–	–
Elemol	1543	1548	–	–	–	1.091	–	–	–	–	–
(E)-Nerolidol	1560	1561	13.03	9.77	–	2.796	–	–	–	–	–
Dodecanoic acid	1565	1565	–	–	–	–	–	–	–	0.082	–
Farnesol < (2E,6Z)- >	1710	1714	3.01	–	0.476	–	–	–	15.604	–	0.164
Epi-bicyclosesquiphellandrene	1518	1520	–	4.12	–	–	–	–	–	–	–
Spathulenol	1574	1577	0.67	–	1.707	–	–	1.24	1.25	–	2.425
Caryophyllene oxide	1578	1582	–	1.83	3.329	–	–	1.881	10.368	–	3.831
Isoaromadendrene epoxide	1580	1579	0.34	–	–	–	–	2.573	–	–	–
Alloaromadendrene oxide	1591	1595	–	–	–	–	–	–	–	–	2.423
Xanthoxylin	1620	1625	1.21	–	7.28	–	–	–	–	–	–
β-Eudesmol	1647	1649	0.87	–	–	–	–	1.683	–	–	2.24
α-Eudesmol	1650	1652	1.12	0.89	4.608	–	–	–	–	–	–
α-Caryophylladienol	1657	1661	1.37	–	5.861	1.023	–	–	–	–	–
1-cyclododecyl-Ethanone	1660	1662	–	–	–	–	–	10.327	–	–	4.673
Valerenol	1690	1699	1.2	–	8.897	0.928	–	–	2.841	–	–
2-Pentadecanone	1692	1697	–	1.39	1.266	2.793	–	–	–	–	–
Diazinone	1740	1745	–	–	1.072	–	–	–	0.832	–	–
2Z,6E-Farnesyl acetate	1818	1821	0.48	2.78	4.75	4.125	–	–	–	–	–
En-in-dicycloether	1900	1902	–	19.97	4.473	47.259	–	–	–	83.6	–
Phytol	1940	1942	–	–	3.07	–	–	–	–	–	–
Hexadecanoic acid	1956	1959	–	–	1.343	–	0.532	–	0.976	–	–
Fluoranthene	2024	2020	–	–	2.4	2.209	–	–	–	–	–
1-decyl-Cyclopentanecarboxylic acid			–	–	–	8.219	–	–	–	6.148	–
Cyclolaurene			–	–	–	4.12	–	–	–	–	–
9,12-Octadecadienoic acid			–	–	1.433	–	–	–	–	–	–
9,12,15-Octadecatrienoic acid			–	–	2.013	–	–	–	–	–	–
Total			81.84	82.27	86.996	90.72	98.703	89.426	89.684	89.911	83.417

^a^Relative retention indices taken from Adams^30^.

E1: *A.tournefortiana* (Kerman province), E2: *A.tournefortiana* (North Khorasan province), E3: *A.tournefortiana* (West Azerbaijan province), E4: *A.khorassanica* (South Khorasan province), E5: *A.khorassanica* (North Khorasan province), E6: *A.khorassanica* (Semnan province), E7: *A.haussknechtii* (Kohgiluyeh and Boyer-Ahmad province), E8: *A.haussknechtii* (Isfahan province), E9: *A.haussknechtii* (Chaharmahal and Bakhtiari province).

82.27% of *A.tournefortiana* essential oil compounds collected from North Khorasan were identified. In this population the most compounds were en-in-dicycloether (19.97%), (*Z*)-nerolidol (15.25%), (*E*)-nerolidol (9.77%) and valencene (9.34%). 49.4% of the identified compounds were sesquiterpenes and 5.17% of the compounds were monoterpenes (Table 1).

For the other population of *A.tournefortiana* was collected from West Azerbaijan 86.99% of total essential oil was identified. (*Z*)-*β*-farnesene (19.46%), valerenol (8.89%), xanthoxylin (7.28%) and *α*-caryophylladienol (5.86%) were the major components. In this ecotype, 59.54% of the identified essential oil compounds were sesquiterpenes and 2.27% of the compounds were monoterpenes (Table 1).

Almost all the compounds (98.70%) in *A.khorassanica* essential oil collected from North Khorasan were identified. The main compounds of oil were camphor (74.22%) and 1,8-cineole (22.91%). 98.06% of the identified essential oil compounds were monoterpenes and other compounds were non-terpenoid (Table 1).

For the other population was collected from South Khorasan 90.72% of total essential oil components were identified. En-in-dicycloether (47.25%), 1-decyclopentanecarboxylic acid (8.21%), (*Z*)-nerolidol (5.24%) and 2*Z*-6*E*-farnesyl acetate (4.12%) were the major components. Polyene were the major components of this essential oil, however, about 26% of the compounds were sesquiterpenes (Table 1).

89.42% of total oil for *A.khorassanica* collected from Semnan were identified. The most compounds were chrysanthenone (16.58%), 1-cyclododecyl-ethanone (10.32%), 1,8-cineole (7.35%) and camphor (5.29%). 58.48% of the compounds identified in the essential oil were monoterpenes and 11.07% of the compounds were sesquiterpenes (Table 2).

**Table 2:** Inhibitory activity of *Artemisia* ecotypes extract.

*Artemisia* ecotypes	Acetyl cholinesterase inhibition (IC_50_ μg/mL)	Tyrosinase inhibition (IC_50_ μg/mL)
E1	310	295
E2	120	110
E3	114	202
E4	284	308
E5	211	104
E6	237	219
E7	201	213
E8	261	253
E9	341	378

E1: *A.tournefortiana* (Kerman province), E2: *A.tournefortiana* (North Khorasan province), E3: *A.tournefortiana* (West Azerbaijan province), E4: *A.khorassanica* (South Khorasan province), E5: *A.khorassanica* (North Khorasan province), E6: *A.khorassanica* (Semnan province), E7: *A.haussknechtii* (Kohgiluyeh and Boyer-Ahmad province), E8: *A.haussknechtii* (Isfahan province), E9: *A.haussknechtii* (Chaharmahal and Bakhtiari province).

For *A.haussknechtii* collected from Kohgiluyeh and Boyer-Ahmad 89.68% of essential oil components were identified. The main components were (2*E*,6*Z*)-farnesol (15.60%), caryophyllene oxide (10.36%), ledene (8.04%) and camphor (7.95%). 43.13% of the identified compounds were sesquiterpenes and 34.90% of essential oil compounds were monoterpenes (Table 1).

About 89.91% of Isfahan's *A.haussknechtii* essential oil components were identified. En-in-dicycloether (83.6%) and 1-decyclopentanecarboxylic acid (6.14%) were main components. In this ecotype, most of the identified essential oil compounds (83.6%) were organic compounds (polyenes) and 0.08% were monoterpenes (Table 1).

For the other population collected from Chaharmahal and Bakhtiari 83.41% of components were identified. 1,8-cineole (16.56%), borneol (16.13%), camphor (11.84%) and 1-cyclododecyl-ethanone (4.67%) were the major components. 60.97% of the essential oil compounds were monoterpenes and 16.17% of the compounds were sesquiterpenes (Table 1).

Some components were just in one population. For example, alloaromadendrene oxide (2.42%) was identified only in the population of Chaharmahal and Bakhtiari population (*A.haussknechtii)*. 9,12-octadecadienoic acid (1.43%), 9,12,15-octadecatrienoic acid (2.01%) and Phytol (3.07%) were identified only in the population of West Azerbaijan (*A.tournefortiana*). Cyclolaurene (4.12%) and elemol (1.09%) were identified only in the population of South Khorasan (*A.khorassanica*). Hydroxy citronellal (2.31%), benzimidazole (4.15%), epi-bicyclosesquiphellandrene (4.12%) were identified only in the population of North Khorasan (*A.tournefortiana*). *δ*-cadinene (2.27%), pulegone (1.51%), cis-carveol (2.07%) and lavandulol (1.09%) were identified only in the population of Kerman (*A.tournefortiana*). (*E*)-methyl isoeugenol (2.11%) and trans-sabinene hydrte (7.59%) were identified only in the population of Kohgiluyeh and Boyer-Ahmad (*A.haussknechtii*). Mesitylene (3.61%), filifolone (4.70%), terpinen-4-ol (2.52%), cyclopentadiene carboxylicacid (5.06%), thymol (2.68%), filifolide A (1.92%) and (*E*)-jasmone (4.86%) were identified only in the population of Semnan (*A. khorassanica*) (Table 1).

The major compounds between the essential oils of ecotypes were en-in-dicycloether, camphor, 1,8-cineole, and (Z)-β-farnesene (Table 1). En-in-dicycloether have shown important insecticidal and acaricidal effects^28^. Camphor has been used in traditional medicine over centuries, probably most commonly as a decongestant. It is used as a topical medication as a skin cream or ointment to relieve itching from insect bites, minor skin irritation, or joint pain. Said to β-farnesene possess DPPH free radical scavenging, anticarcinogenic, antibacterial, and antifungal activity and it also had demonstrated dose-related neuroprotective effects on cultured rat primary cortical neurons, blocking H_2_O_2_-induced intracellular LDH release and reduced DNA damage 47.8%, suggesting application in neurodegenerative diseases^29^.

**Figure 2:**
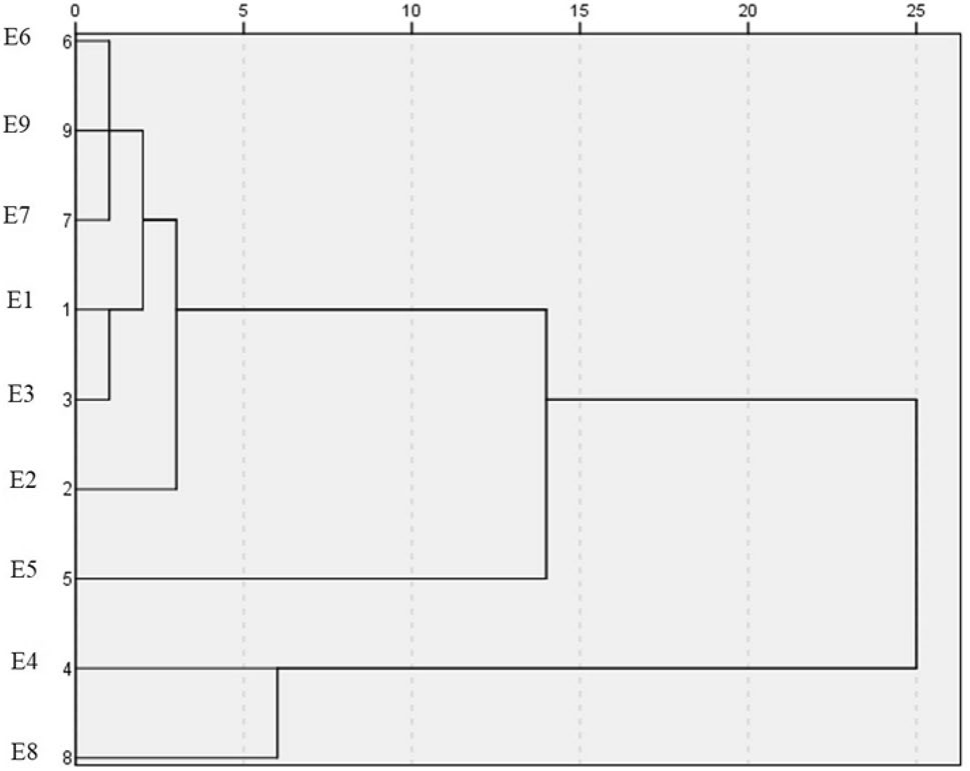
Dandrogram of essential oil compounds cluster analysis. E1: *A.tournefortiana* (Kerman province), E2: *A.tournefortiana* (North Khorasan province), E3: *A.tournefortiana* (West Azerbaijan province), E4: *A.khorassanica* (South Khorasan province), E5: *A.khorassanica* (North Khorasan province), E6: *A.khorassanica* (Semnan province), E7: *A.haussknechtii* (Kohgiluyeh and Boyer-Ahmad province), E8: *A.haussknechtii* (Isfahan province), E9: *A.haussknechtii* (Chaharmahal and Bakhtiari province).

Based on cluster analysis of essential oils compounds the investigated populations were divided into three different clusters. Isfahan's (*A.haussknechtii*) and South Khorasan's (*A. khorassanica*) populations had the highest amounts of en-in-dicycloether (47–83%) and 1-decyl-Cyclopentanecarboxylic acid (6.1–8.2%) and placed in a separate cluster. Interestingly these components have been identified for the first time as the main compounds of the species. North Khorasan's population (*A.Khorassanica*) with highest amount of camphor placed in a distinct cluster. Previously, camphor has been reported as one of the major components in *A. khorassanica*, which was collected from the north of Iran and Khorasan^22^^,^^23^. The rest of the populations placed in a separate cluster (Fig. 2).

#### TPC

**Figure 3:**
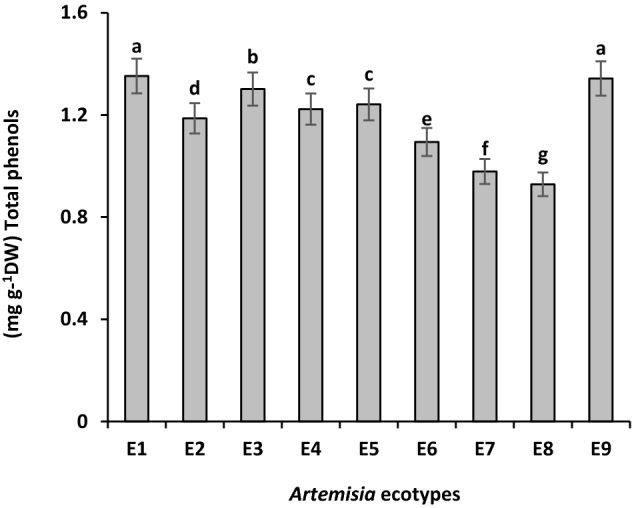
Comparison of mean total phenol ± standard error in different *Artemisia* ecotypes. E1: *A.tournefortiana* (Kerman province), E2: *A.tournefortiana* (North Khorasan province), E3: *A.tournefortiana* (West Azerbaijan province), E4: *A.khorassanica* (South Khorasan province), E5: *A.khorassanica* (North Khorasan province), E6: *A.khorassanica* (Semnan province), E7: *A.haussknechtii* (Kohgiluyeh and Boyer-Ahmad province), E8: *A.haussknechtii* (Isfahan province), E9: *A.haussknechtii* (Chaharmahal and Bakhtiari province).

*A. tournefortiana* which collected from Kerman with 1.35 ± 0.01 mg g^−1^ DW and *A. haussknechtii* collected from Chaharmahal and Bakhtiari province with 1.34 ± 0.005 mg g^−1^ DW had the highest amount of total phenol content compared with other regions. After them, *Artemisia* of West Azerbaijan region (*A. tournefortiana*) with 1.30 ± 0.0 mg g^−1^ DW had more total phenol. Then the highest amount of total phenol belongs to *A. khorassanica* collected from North Khorasan and South Khorasan and they did not differ significantly. After them, total phenol of *A. tournefortiana* collected from North Khorasan with 1.18 ± 0.001, Semnan's *A. khorassanica* with 1.09 ± 0.007 and *A. haussknechtii* collected from Kohgiluyeh and Boyer-Ahmad province with 0.97 ± 0.003 mg g^−1^ DW was higher. The lowest amount of phenolic compounds belongs to Isfahan region (*A. haussknechtii*) with 0.92 ± 00.7 mg g^−1^ DW (Fig. 3). Previous study on* A. biennis* Willd, showed that, the hydroethanolic extract of the plant had the highest amount of phenolic content and antioxidant activity^31^. Another research on *A. absinthium* demonstrated that the ethanolic extract had more TPC than *A. dracunculus* and *A. annua*^32^. In another study, among six *Artemisia* species, *A. oliveriana* had the highest TPC and *A. diffusa* had the highest TFC^33^.

#### TFC

**Figure 4:**
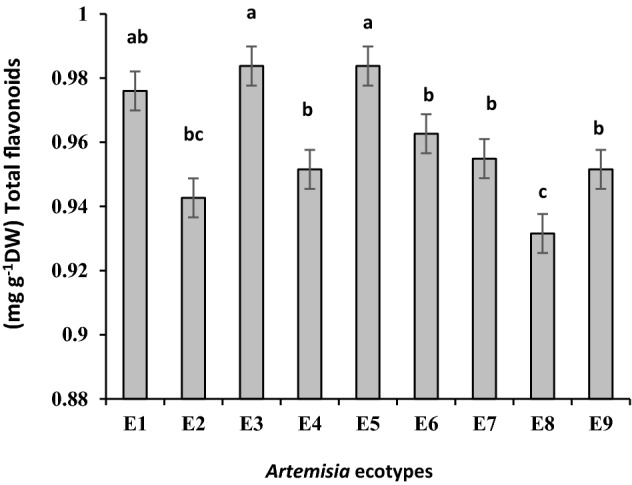
Comparison of mean total flavonoid ± standard error in different *Artemisia* ecotypes. E1: *A.tournefortiana* (Kerman province), E2: *A.tournefortiana* (North Khorasan province), E3: *A.tournefortiana* (West Azerbaijan province), E4: *A.khorassanica* (South Khorasan province), E5: *A.khorassanica* (North Khorasan province), E6: *A.khorassanica* (Semnan province), E7: *A.haussknechtii* (Kohgiluyeh and Boyer-Ahmad province), E8: *A.haussknechtii* (Isfahan province), E9: *A.haussknechtii* (Chaharmahal and Bakhtiari province).

*A. tournefortiana* which collected from West Azerbaijan with 0.98 ± 0.01 and *A. khorassanica* collected from North Khorasan with 0.98 ± 0.009 mg g^−1^ DW had the highest total flavonoids. After them, Kerman's *A. tournefortiana* with 0.97 ± 0.012 mg g^−1^ DW had higher total flavonoids and the Isfahan's ecotype (*A. haussknechtii*) with 0.93 ± 0.01 mg g^−1^ DW had the lowest total flavonoids (Fig. 4).

### Antioxidant activity (DPPH)

**Figure 5:**
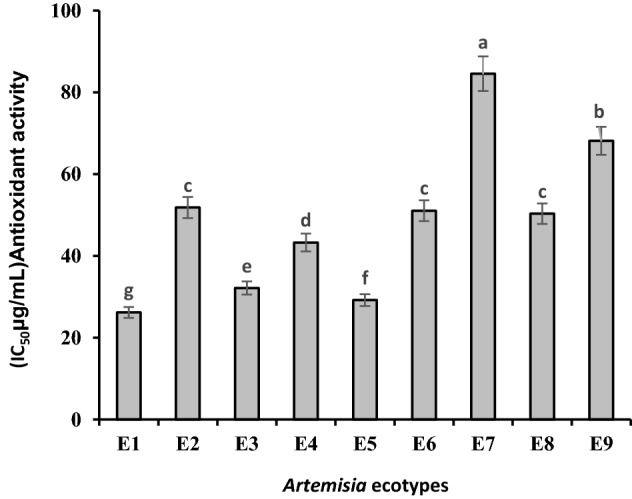
Comparison of mean ± standard error of antioxidant activity of *Artemisia* ecotypes by DPPH method. E1: *A.tournefortiana* (Kerman province), E2: *A.tournefortiana* (North Khorasan province), E3: *A.tournefortiana* (West Azerbaijan province), E4: *A.khorassanica* (South Khorasan province), E5: *A.khorassanica* (North Khorasan province), E6: *A.khorassanica* (Semnan province), E7: *A.haussknechtii* (Kohgiluyeh and Boyer-Ahmad province), E8: *A.haussknechtii* (Isfahan province), E9: *A.haussknechtii* (Chaharmahal and Bakhtiari province).

Antioxidant activity of methanolic extract by DPPH method was higher with 26.17 ± 0.03 μg/mL in plants collected from Kerman (*A.* tournefortiana) than other regions. After that, North Khorasan's *A. khorassanica* with 29.19 ± 0.07, West Azerbaijan's *A. tournefortiana* with 32.14 ± 0.46, and South Khorasan's *A. khorassanica* with 43.28 ± 0.09 μg/mL had higher antioxidant activity. Then, the ecotypes belonging to North Khorasan (*A. tournefortiana*), Semnan (*A. khorassanica*) and Isfahan (*A. haussknechtii*) showed good antioxidant activity that did not differ significantly. Also, the ecotype belonging to Kohgiluyeh and Boyer-Ahmad (*A. haussknechtii*) with 68.13 ± 0.50 μg/mL showed the least activity (Fig. 5).

### Antioxidant activty (FRAP)

**Figure 6:**
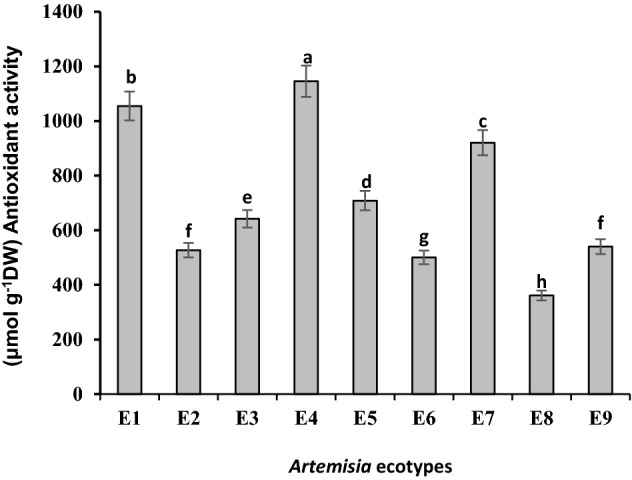
Comparison of mean ± standard error of antioxidant activity by FRAP method. E1: *A.tournefortiana* (Kerman province), E2: *A.tournefortiana* (North Khorasan province), E3: *A.tournefortiana* (West Azerbaijan province), E4: *A.khorassanica* (South Khorasan province), E5: *A.khorassanica* (North Khorasan province), E6: *A.khorassanica* (Semnan province), E7: *A.haussknechtii* (Kohgiluyeh and Boyer-Ahmad province), E8: *A.haussknechtii* (Isfahan province), E9: *A.haussknechtii* (Chaharmahal and Bakhtiari province).

Antioxidant activity of methanolic extracts by FRAP method was higher with 1145.69 ± 3.38 μmol g^−1^ DW in plants collected from South Khorasan (*A. khorassanica*) than other regions. Then, Kerman's *A. tournefortiana* with 1054.94 ± 4.42, *A. haussknechtii* collected from Kohgiluyeh and Boyer-Ahmad with 920.74 ± 19.67, North Khorasan's *A. khorassanica* with 708.57 ± 5.57, and West Azerbaijan's *A. tournefortiana* with 642.11 ± 10.46 μmol g^−1^ DW had more activity, respectively. After them, antioxidant activity was high in *A. haussknechtii* collected from Chaharmahal and Bakhtiari, and North Khorasan's *A. khorassanica* that didn't differ significantly. Semnan's *A. khorassanica* activity was 500.24 ± 3.38 μmol g^−1^ DW. The lowest level of antioxidant activity was observed in Isfahan ecotype (*A. haussknechtii*) with 360.93 ± 3.83 μmol g^−1^ DW. (Fig. 6). Simple correlation analysis showed a significant relationship between the TFC and TPC content (r = 0.61, *p* < 0.05). Furthermore, correlation analysis revealed that 1,8-cineole content is correlated with camphor (r = 0.86, *p* < 0.01). Moreover, there were negative correlations between en-in-dicycloether and TFC content (r = -0.67*, p-value* < 0.05). However, there was no any significant correlation between antioxidant activity and essential oil components (Table 3).

**Table 3:** Correlation coefficients among antioxidants and major essential oil components of *Artemisia* accessions.

	FRAP	DPPH	TFC	TPC	(Z)-β-farnesene	1,8-cineole	Camphor	En-in-dicycloether
FRAP	1.00							
DPPH	− 0.16	1.00						
TFC	0.31	− 0.6	1.00					
TPC	0.31	− 0.52	0.61*	1.00				
(Z)-β-farnesene	0.07	− 0.48	0.58	0.44	1.00			
1,8-cineole	− 0.22	− 0.02	0.36	0.28	− 0.35	1.00		
camphor	0.01	− 0.31	0.53	0.2	− 0.21	0.83**	1.00	
En-in-dicycloether	− 0.2	− 0.02	− 0.67*	− 0.52	− 0.24	− 0.42	− 0.33	1.00

*Correlation is significant at the 0.05 level.

**Correlation is significant at the 0.01 level.

### Acetyl cholinesterase inhibitory activity

According to Table 2, the extract of *A. tournefortiana* species belonging to West Azerbaijan with 114 μg/mL had the highest inhibitory activity of acetylcholinesterase. Then, North Khorasan's *A. tournefortiana* with 120, *A, haussknechtii* collected from Kohgiluyeh and Boyer-Ahmad with 201, North Khorasan's *A. khorassanica* with 211, and Semnan's *A. khorassanica* with 237 μg/mL had highest acetyl cholinesterase inhibitory activity, respectively. After these, Isfahan's *A. haussknechtii* with 261, South Khorasan's *A. khorassanica* with 284, and Kerman's *A. tournefortiana* with 310 μg/mL had more inhibitory activity. The lowest inhibitory activity was observed in *A. haussknechtii* collected from Chaharmahal and Bakhtiari with 341 μg/mL. Previous study showed that methanolic extract of *A. asiatica* showed the highest inhibition of acetylcholinestersa among extracts of various plants^34^.

### Tyrosinase inhibitory activity

According to Table 2, the extract of *A. khorassanica* species belonging to North Khorasan had the highest tyrosinase inhibitory activity with 104 μg/mL. After that, ecotypes of North Khorasan (*A. tournefortiana*) with 110, West Azerbaijan (*A. tournefortiana*) with 202, Kohgiluyeh and Boyer-Ahmad (*A. haussknechtii*) with 213, and Semnan (*A. khorassanica*) with 219 μg/mL had highest activity, respectively. Then, Isfahan's *A. haussknechtii* with 253, Kerman's *A. tournefortiana* with 295, and South Khorasan's *A. khorassanica* with 308 μg/mL had more inhibitory activity. *A. haussknechtii* collected from Chaharmahal and Bakhtiari also had the lowest tyrosinase inhibitory activity (IC_50_: 378 μg/mL). According to the study on ethanolic extract of *A. iwayomogi,* it has been found that it has a high antioxidant activity (by DPPH) and high tyrosinase inhibitory activity^35^. Another study found that microwave assisted extraction of *A. pallens* had a higher tyrosinase inhibitory effect than soxhlet extraction^36^.

## Conclusions

There were variations in main components of essential oil among species and ecotypes. These variations are probably related with different environmental conditions of the plants. So, due to the various compounds in essential oils, different ecotypes can be used in different industries. Among the ecotypes of *A. tournefortiana*, the ecotype belonging to Kerman had the highest essence yield and the ecotype of North Khorasan had the lowest yield. Among the ecotypes of *A. khorassanica* species, the ecotypes belonging to the North and South Khorasan had the highest and the lowest essential oil yields, respectively. There was not much difference between the essential oil yields of *A. haussknechtii* ecotypes, but essence yield of Kohgiluyeh and Boyer-Ahmad ecotype was slightly lower than the others. Isfahan's *A. haussknechtii* methanolic extract had the lowest TFC, TPC, and antioxidant activity with FRAP method. The lowest level of antioxidant activity with DPPH method, was in *A. haussknechtii* collected from Kohgiluyeh and Boyer-Ahmad. Kerman's *A. tournefortiana* methanolic extract had the most TPC and antioxidant activity by DPPH method. *A. haussknechtii* collected from Chaharmahal and Bakhtiari, similar to the Kerman's *A. tournefortiana* had the most TPC, but the latter ecotype had the lowest tyrosinase and acetyl cholinesterase inhibition. *A. khorassanica* collected from North Khorasan had the most TFC and the highest tyrosinase inhibition, but the one collected from South Khorasan had the most antioxidant activity with FRAP method. West Azerbaijan's *A. tournefortiana* similar to the North Khorasan's *A. khorassanica* had the most TFC and the highest acetyl cholinesterase inhibition. Thus, these two species are the superior species with the best medicinal value, which can be introduced for cultivation in different regions of Iran and other regional countries.

## Data Availability

The datasets used and/or analyzed during the current study available from the corresponding author on reasonable request.

## Abbreviations

GC–MS: Gas chromatography–mass spectrometry
Nrf2: Nuclear factor-erythroid factor 2-related factor 2
AchEI: Acetyl-cholinesterase-inhibitor
AD: Alzheimer's disease
AchE: Acetyl-cholinesterase
TFC: Total flavonoid content
TPC: Total phenolic content
IBRC: Iranian biological resource center
NIST: National institute of standards and technology
DPPH: 2,2-Diphenyl-1-picrylhydrazyl
FRAP: Fluorescence recovery after photobleaching
TPTZ: Tripyridyltriazine
DTNB: Dithionitrobenzoic acid
DMSO: Dimethyl sulfoxide
LDH: Lactate dehydrogenase
DNA: Deoxyribonucleic acid
